# Poisoning by Bad Meat

**Published:** 1890-05-03

**Authors:** 


### POISONING BY BAD MEAT.

Last summer, in the camp at Avors, during the annua manœuvres, no fewer than 227 French soldiers, who had partaken of meat in a state of incipient decomposition, were attacked with vomiting, diarrhœa, vertigo, profuse sweating, and general prostration. A peculiar symptom in addition was marked and prolonged dilatation of the pupils. These lasted from six days to three weeks, but one man died on the fourteenth day.

